# Multimodality Molecular Imaging of Stem Cells Therapy for Stroke

**DOI:** 10.1155/2013/849819

**Published:** 2013-10-08

**Authors:** Fangfang Chao, Yehua Shen, Hong Zhang, Mei Tian

**Affiliations:** ^1^Department of Nuclear Medicine, Second Affiliated Hospital of Zhejiang University School of Medicine, 88 Jiefang Road, Hangzhou, 310009 Zhejiang, China; ^2^Zhejiang University Medical PET Center, Zhejiang University, Hangzhou, China; ^3^Institute of Nuclear Medicine and Molecular Imaging, Zhejiang University, Hangzhou, China; ^4^Key Laboratory of Medical Molecular Imaging of Zhejiang Province, Hangzhou, China

## Abstract

Stem cells have been proposed as a promising therapy for treating stroke. While several studies have demonstrated the therapeutic benefits of stem cells, the exact mechanism remains elusive. Molecular imaging provides the possibility of the visual representation of biological processes at the cellular and molecular level. In order to facilitate research efforts to understand the stem cells therapeutic mechanisms, we need to further develop means of monitoring these cells noninvasively, longitudinally and repeatedly. Because of tissue depth and the blood-brain barrier (BBB), in vivo imaging of stem cells therapy for stroke has unique challenges. In this review, we describe existing methods of tracking transplanted stem cells in vivo, including magnetic resonance imaging (MRI), nuclear medicine imaging, and optical imaging (OI). Each of the imaging techniques has advantages and drawbacks. Finally, we describe multimodality imaging strategies as a more comprehensive and potential method to monitor transplanted stem cells for stroke.

## 1. Introduction

Stroke remains the third-leading cause of death and the main cause of disability worldwide, leaving a significant socioeconomic burden [1]. With the intensification of aging, the prevalence of stroke and the costs associated with caring for afflicted patients are likely to increase substantially in the coming years. Ischemia stroke caused by the occlusion of a cerebral artery represents the most common kind of a cerebrovascular event and accounting for 85–90% of all incidences [1]. Despite numerous studies on neuroprotective agents, the only effective and FDA approved therapy for ischemic stroke is the removal of the thrombus/embolus either pharmacologically by thrombolysis with recombinant tissue plasminogen activator, which has a very short therapeutic time window of 4–6 h after onset of ischemia [2] or mechanically with a retriever [3]. However, those approaches for stroke are limited to acute measures designed to restore perfusion and to protect ischemic cells from death in the acute phase, which are unable to replace damaged or lost neural cells [4]. Up till now, no effective treatment to improve functional recovery exists in the subacute or chronic phase. 

Alternatively, cell-based therapy is emerging as a promising new modality for enhancing tissue repair and neurologic recovery in ischemic stroke [5]. The main objective of cell-based therapies is to repopulate the damaged tissue with functional cells, with the final goal that these cells will integrate with the remaining functional native cells and contribute to the recuperation of the lost organ function. However, more recent studies indicate that stem cells instead can enhance functional recovery poststroke via alternative mechanisms, including stem cells secretion of neurotrophic factors, immunomodulation, stimulation of endogenous neurogenesis, and neovascularization [6–8]. Therefore, the underlying mechanisms of the benefit in functional performance remain under dispute, and the fate of the transplanted stem cells including survival, death, proliferation, migration, or differentiation is multiple [9]. In addition, stem cell-based therapy has to deal with the possible complication of spontaneous tumor formation. It is, therefore, necessary to carefully monitor transplant dynamics over a long period of time. Animal experiments previously have assessed in the fate of injected stem cells through ex vivo methods on sacrificed animals, which is restricted to one specific time point, and many animals are needed to assess the whole temporal dynamics of a graft. For clinical use, it would be of tremendous value to develop multimodal, noninvasive, sensitive, and in vivo imaging approaches to track transplanted cells, to monitor their survival and immune reaction, to monitor their migration and proliferation, as well as the therapeutic response in the living subjects, and to confirm the transplantation parameters such as optimum time for surgery after the insult, cell type and source, and the optimal route of cell delivery. 

In order to develop successful in vivo imaging modalities for transplanted stem cells, several factors must be taken into consideration. For one thing, it should have high spatial resolution and high sensitivity so as to detect even the minuscule signals. The technology would also not impact cellular viability, motility, differentiation, physiology, or functionality. In addition, the signal strength would be specific for and corroborate cell viability, and not to be present when the cell is dead. Furthermore, virtually all stem cells imaging technologies require some degree of ex vivo alteration of the stem cells prior to implantation of the cells, and so efficiency of alteration is also a requirement. Since these techniques will be used to image cells within the brain, any potential probes must be capable of crossing the blood-brain barrier (BBB).

Current imaging modalities have been proposed for in vivo tracking of stem cells transplantation for stroke, including magnetic resonance imaging (MRI), nuclear medicine imaging and optical imaging (OI). In this paper, we will review each of these in vivo imaging techniques and compare the benefits and drawbacks of each approach. What is more, we describe multimodality molecular imaging of stem cells transplantation because each technique has advantages and disadvantages. Combination of two or more methods into a multimodal approach holds the opportunity to look at comprehensive aspects of a stem cell graft, provided by the different imaging modalities, within the same animal. Integration of these pieces of information will allow for a more stringent control over the cell grafts and elucidate the mechanisms of the action of stem cells therapy in cerebral ischemia.

## 2. Magnetic Resonance Imaging 

MRI is a widely used medical imaging technique in which magnetic fields are used to detect the nuclear spin of molecules. Its noninvasive nature makes longitudinal studies possible, allowing for repetitive measurements on the same animal and thus producing the full temporal profile of mechanistic events to each individual. Furthermore, it will reduce the need to sacrifice whole groups at different survival times, which is essential to transfer to the clinical patients. It has an excellent soft-tissue contrast with high spatial resolution down to 50 *μ*m or even single cell detection [10], which makes it to be used in cell tracking ideally. MRI has been successfully applied to detect and track stem cells migration, particularly in cardiac [11, 12] and brain disease [13, 14] models.

This imaging modality involves preimplantation ex vivo enrichment of the stem cells with a contrast agent to produce a positive or negative signal that allows the cells to be distinguished from the background once implanted in the brain. Typically, contrast agents can be divided into paramagnetic (gadolinium-based or manganese-based) or superparamagnetic (iron oxide-based) agents, but other nuclei, such as ^19^F, can also be used to monitor transplanted cells. Superparamagnetic and paramagnetic contrast agents are not detected directly, but instead through their effects on the intrinsic ^1^H signal (i.e., their effect on local water relaxation rates) [15]. ^19^F-MRI of cells labeled with fluorinated contrast agents does not use an indirect effect like modulation of T1 or T2/T2* relaxation but measures the ^19^F signal directly using spin density-weighted MRI or MR spectroscopy [15]. 

Initially, the contrast agent gadolinium rhodamine dextran (GRID) that induces a positive or white contrast, appearing as hyperintense signals on T1-weighted MR images was used to track human neural stem cells (hNSCs) in the stroke-lesioned brain, demonstrating the feasibility of cellular MRI to monitor cell migration in vivo. However, a recent study indicated the deleterious effects of GRID-based contrast agents on long-term in vivo functional properties of NSCs grafted in the stroke-injured rodent brain [16]. 

Alternatively, iron oxide nanoparticles cause the adjacent protons to experience a large dipolar magnetic field gradient and thereby detecting via magnetic resonance as hypointense (black) contrast on T2- and T2*-weighted magnetic resonance [17, 18]. Iron oxide nanoparticles which usually are comprised of a mixed-valence (Fe^2+^ and Fe^3+^) iron core with varying chemical coatings, such as dextran, can be categorized according to size [18]. Superparamagnetic iron oxide particles (SPIO) range in size from 50 to 200 nm in diameter. Ultrasmall superparamagnetic iron oxide particles (USPIO) measure less than 50 nm in diameter and micron-sized paramagnetic iron oxide particles (MPIO) measure on the order of 1 *μ*m in diameter. The most studied and preferred contrast agent is SPIO, which is degraded safely along physiologic iron metabolism pathways, and some SPIOs are already US FDA approved for use in humans [19]. 

MRI using SPIO has the advantage of superior 3D spatial resolution but unfortunately has poor sensitivity, detecting molecules at the millimolar to micromolar level [20]. Furthermore, hypointensity on T2 and T2* can also be due to a number of false positives, including bleeds, air, hemorrhagic transformation of stroke lesions, and macrophage phagocytosis of endogenous iron as a part of the inflammatory response to certain pathologies [21, 22]. The concentration of SPIO in stem cells is halved with cell division and thus the signal will degrade with in vivo cellular proliferation [23, 24]. Furthermore, since contrast is achieved indirectly through disturbances of the local magnetic field experienced by the surrounding hydrogen nuclei, quantification of the number of cells in vivo is questionable [15, 25].

Hoehn et al. [26] were the first to use iron oxide nanoparticles to track the transplanted stem cells in vivo over 3-4 weeks in a rat model of middle cerebral artery occlusion (MCAO). Murine embryonic stem cells (ESCs) were labeled with USPIO and transplanted at 2 weeks after stroke into the contralateral cortex and striatum. During 3 weeks, cells migrated along the corpus callosum to the ventricular walls and massively populated the border zone of the damaged brain tissue on the hemisphere opposite to the implantation sites. The authors confirmed the in vivo findings by immunohistochemical staining for green fluorescent protein (GFP), which was stably expressed by the transplanted cells. Their results indicated that ESCs cells had high migrational dynamics, targeted to the cerebral lesion area. MRI is suited for the noninvasive observation of cell migration, engraftment, and morphological differentiation at high spatial and temporal resolution. 

**Figure 1:**
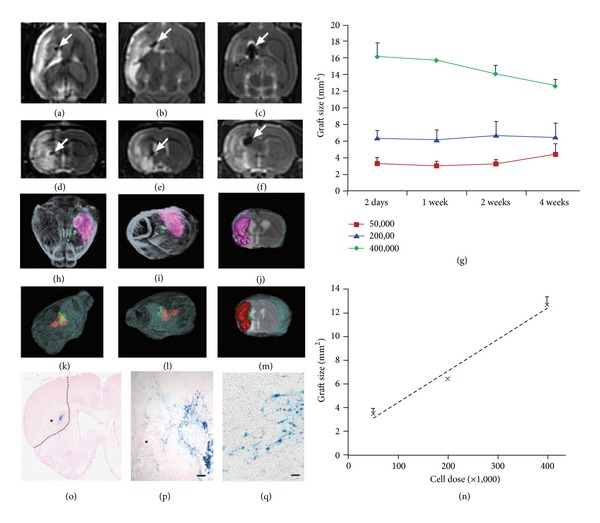
MRI imaging analysis of hNSC grafts in an experimental stroke model. (a–c) MRI horizontal and (d–f) frontal scans show dose-dependent size of the SPIO-labeled hNSC grafts as hypointense areas in the striatum (arrow) and medially in the penumbral zone of the stroke region distinguished as strongly hyperintense areas on T2-weighted images. The cells doses are 50,000 cells (a, d), 200,000 cells (b, e), and 400,000 cells (c, f). (g) Quantitative analysis of graft size, in consecutive coronal MRI scans, 600 *μ*m spaced, in the 3 animal groups (*N* = 15) over the posttransplant survival time confirms the BLI data and shows a stable graft size demonstrating survival of the graft. Three-dimensional surface rendering reconstruction of grafted rat brain from high-resolution T2-MRI illustrates the grafts (green) and stroke (pink, red) in a representative animal from the (h–j) low dose and (k–m) intermediate dose group. (n) The MRI measured graft size shows a strong correlation (*R* ^2^ = 0.99) with the cell dose transplanted. (o–q) Histological analysis using prussian blue staining for SPIO particles demonstrates the cytosolic deposition of blue crystals in the grafted hNSCs and migration of hNSCs towards the stroke area (asterisks in o, p). Interrupted line in (o) shows the boundary of the stroke zone. Scale bars = (p) 50 *μ*m; (q) 20 *μ*m.

 In another study, Daadi et al. [9] investigated the fate of grafted SPIO labeled hNSCs and derived from human ESCs in a stroke environment over time and the fidelity of MRI to detect dose-dependent effects of the grafts on the lesion. MRI analysis clearly detected the grafts as hypointense areas in the striatum and the stroke zone as a hyperintense region in the striatum and cortex on T2-weighted images (Figure 1). The graft size for each analysis in the serial MRI scans demonstrated a linear correlation between the injected cell dose and the MRI size of the transplant. Three-dimensional reconstructions of the grafts and stroke by surface rendering from the MR scans allowed for an accurate representation of both the graft and the stroke sizes and visualized the relationship between small and large grafts to stroke region. Histopathological examinations with prussian blue staining for iron, immunohistochemistry, and confocal analyses demonstrated the survival and migration of grafted SPIO-labeled hNSCs towards the ischemic region.

MRI has even been used in human clinical cases of stem cells transplantation in the brain. In one case of Zhu et al. [27], a patient with traumatic brain injury had exposed neural tissue collected and cultured to obtain a sample of NSCs that were subsequently labeled using SPIO. The cells were then implanted stereotactically near the region of brain damage and followed with T2-weighted MRI every week for 10 weeks. Subsequently, the hypointense signal at each injection site faded but intensified around the lesion. The hypointense signal generated by the cells demonstrated movement of the cells from the implantation site to the periphery of the lesion as early as the first week. However, the hypointense signal disappeared by the seventh week, which the group attributed to NSCs proliferation.

The adult brain executes a continuous generation of endogenous stem cells in the subgranular zone (SGZ) of the hippocampus and the subventricular zone (SVZ) along the lateral ventricle. Within the SVZ, dividing stem cells gives rise to neural progenitor cells (NPCs) [28], which normally travel along the rostral migratory stream to the olfactory bulb where they differentiate into inhibitory interneurons [29]. MRI has recently been used to monitor the migration of endogenous stem cells from the SVZ to the olfactory bulb in animal models [30, 31]. Injection of MPIO into the lateral ventricle resulted in an uptake of the particles by all the cells surrounding the ventricle. Amongst these were the endogenous stem cells and the early NPCs, which would migrate out of the unspecifically labeled territory and along the rostral migratory stream towards the olfactory bulb. These cells have now been imaged with MRI [30] and the rostral migratory path was visible as a hypointense track on the MR images [30, 31].

**Figure 2:**
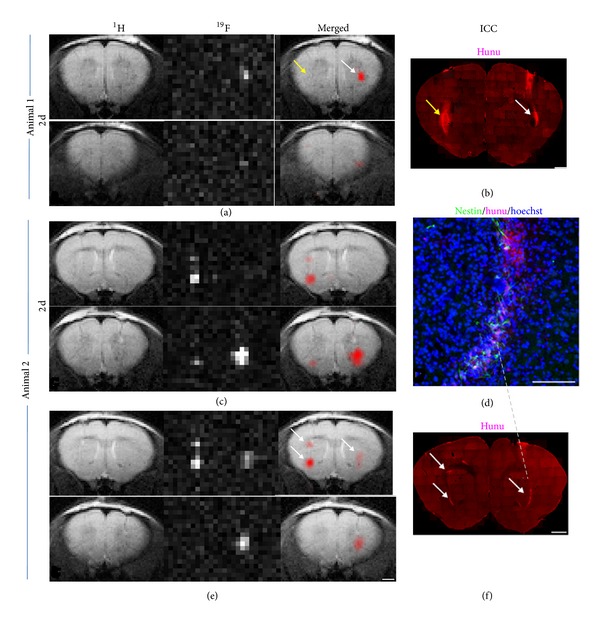
In vivo  ^19^F MRI and correlation with immunohistochemistry. ^1^H, ^19^F, and merged MR images of a mouse (animal 1), which had been injected with nonlabeled control cells into the left striatum and labeled NSCs into the right hemisphere (a). Only the labeled cells generated a ^19^F signal, whereas hunu staining confirmed the presence of cell grafts on both sides as indicated by the arrows (b). MRI of another mouse (animal 2) 2 days (c) and 6 days (e) after grafting showed no major signal loss in the ^19^F images over time. This animal had received two deposits of labeled cells in the left striatum and one deposit in the right striatum. The location and intensity of ^19^F signal from cell clusters, marked with white arrows, correlated well with hunu staining on histological sections. Note that the ^19^F resolution allows the distinction of the two clusters on the left hemisphere (b, f). Only cells that were clearly immunoreactive to both hoechst and hunu were considered as grafted human NSCs (d). Scale bars are 50 mm for (d) and 1 mm for all others.

Unfortunately, the contrast generated by ironoxide labeled cells is neither specific due to ambiguous background nor quantitative. A strategy to overcome these drawbacks is ^19^F MRI of cells labeled with perfluorocarbons [32–34]. The ^19^F nucleus is particularly suitable for labeling as it has a natural abundance of 100%, it is similarly sensitive as ^1^H MRI (83% of ^1^H), and the chemical integrity of the ^19^F-based contrast agent can be validated by ^19^F MR spectroscopy noninvasively [25]. Furthermore, the signal intensity is directly proportional to the number of accumulated ^19^F, hence allowing in vivo quantification of ^19^F labeled cells. In addition, since the level of background ^19^F signal in host tissue is virtually absent, overlaying the ^19^F image on an anatomical ^1^H image allows for unambiguous, quantitative tracking of labeled cells in vivo. One study of tracking hNSCs with ^19^F by Boehm-Sturm et al. [32] showed that ^19^F MRI is a useful tool for the longitudinal tracking of implanted cells in the brain (Figure 2). They found that NSCs could be efficiently labeled with ^19^F with little effects on viability or proliferation and differentiation capacity. However, compared to labeling and tracking with metal-based contrast agents, the technique was considerably less sensitive, requiring a large amount of ^19^F to accumulate in order to generate sufficient signal-to-noise ratio. Therefore, this technique is still in its infancy and further study into the possibility of is needed to determine if such an approach could be used to track stem cells transplanted into the stroke injured brain.

**Figure 3:**
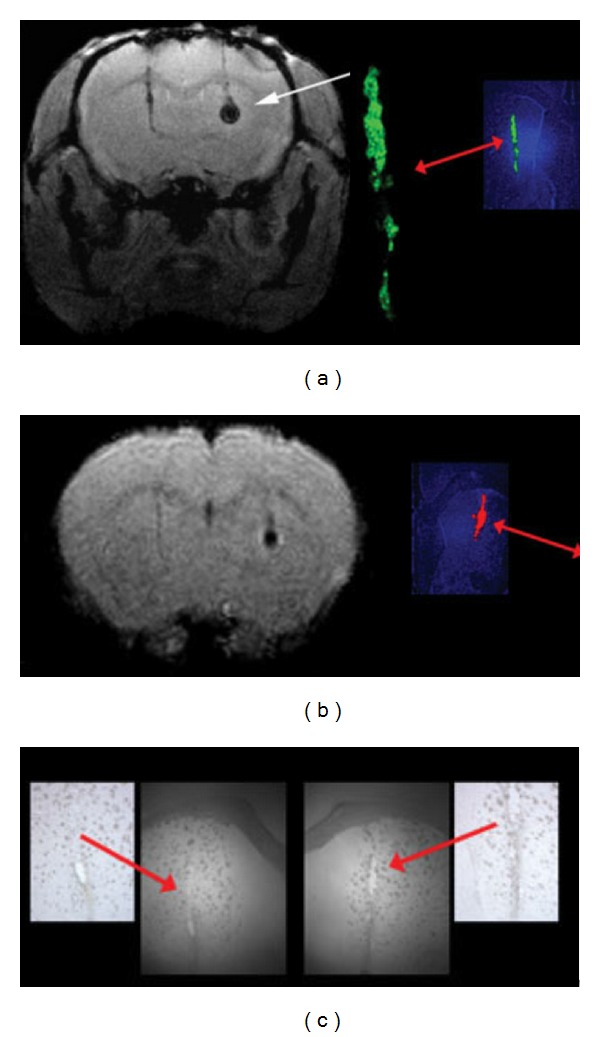
MRI of magA cell induction in vivo. (a) T2*-weighted image of a mouse brain with transplanted magA cells (right, white arrow) and GFP control cells (left). These cells were not induced or incubated with iron supplement prior to transplantation. MagA cells exhibited significantly lower MR signals, reflecting an increase in R2 and suggesting that magA cells are able to use endogenous iron sources. The control cells on the left do not show this effect. (b) MR image of the same mouse brain showing that magA cells are readily seen by T2WI, which is less sensitive than T2* for magnetic nanoparticles. Fluorescence histology confirmed the presence of control (green) and magA-positive (red) cells. Magnified green and red channels are also shown. (c) Brightfield histology section of the brain, indicating the site of the transplanted cells.

Other than contrast agents, magnetic resonance reporter genes are another possible means of magnetic resonance labeling NSCs. While this technique is still in its infancy, some groups have already demonstrated the ability to transfect cells with genes coding for heavy- and light-chain ferritin which is recognized as a storage pool of iron [35]. Over expression of ferritin augments iron uptake and then allows the cells to be visualized by magnetic resonance. Use of magnetic resonance reporter genes would circumvent the problem of contrast agent dilution with cellular proliferation. However, it should be noted that these reporters must be controlled by conditional regulation so as to prevent iron-overload toxicity. Therefore, further study into the possibility of magnetic resonance reporter genes is needed before this technology can be used for stem cells transplantation into the stroke injured brain [36]. Recently, the MagA gene, which synthesizes magnetosomes in some bacteria, has attracted research attention. The gene product has properties in common with SPIO nanoparticles. In a study by Zukiya et al., the T2*-weighted images of magA-positive cells showed significant signal drops as compared to those of control cells. Electron microscopy revealed uniform particles magnetosomes. Cytosolic G6PD and MTT assays did not show cytotoxicity or change in cell proliferation. Particles produced in the magA-transfected animal cell do not cause immune response trigger on cell surfaces (Figure 3). Accordingly, it has been suggested that magA-based imaging may provide a new means of noninvasive cellular tracking in the brain [37].

## 3. Nuclear Medicine Imaging 

Nuclear medicine imaging techniques, including single photon-emission computed tomography (SPECT), and positron emission tomography (PET), represent another promising imaging modality to track stem cells in vivo. Nuclear medicine imaging involves imaging radiotracers, which can bind to different ligands. A main advantage of PET and SPECT is the extreme sensitivity of these imaging techniques, which detect molecules at the nanomolar level [38]. There are two main strategies for in vivo stem cells detection using nuclear imaging: direct imaging and indirect imaging.

Direct imaging which is direct labels cell with a radioactive probe has been used for many years to track cells in vivo. Cells are incubated with a radiotracer, which allows lipophilic molecules to diffuse and be “trapped” in the cells. After a short incubation period, the cells are washed to remove any unbound activity and then injected into the host. Labeling implanted cells with relatively long-lived isotopes allows short-term, real-time cell tracking. Radioisotopes, such as ^111^In (for SPECT) and ^64^Cu (for PET), have long half-lives (67 h and 12.7 h, resp.) and, therefore, can assess the biodistribution of transplanted cells straight after injection [39–41]. In one study of Gleave et al. [42], they developed a method to label neural stem and progenitor cells with ^99m^Tc to visualize these cells in the brain with SPECT. The cells were initially labeled with a permeation peptide carrying a chelator for ^99m^Tc. The accuracy of the transplant location obtained by SPECT was confirmed by comparison with phosphor images and histologic sections of the brain. However, the labeling decreased the proliferative capacity of the neural stem and progenitor cells. The labeling technique described here can be used to standardize the location of cell transplants in the brain and quantify the number of transplanted cells. This information will be useful in standardizing cellular transplantations to achieve reproducible outcomes [42]. Labeling of cells with exogenous nonspecific nuclear imaging agents is easily achievable. However, there are several disadvantages to direct imaging, including the leakage of radionuclides into nontarget cells, limited time window for imaging due to half-life decay [43], dilution of signal from cell division, and lack of ability to determine cell viability and function.

**Figure 4:**
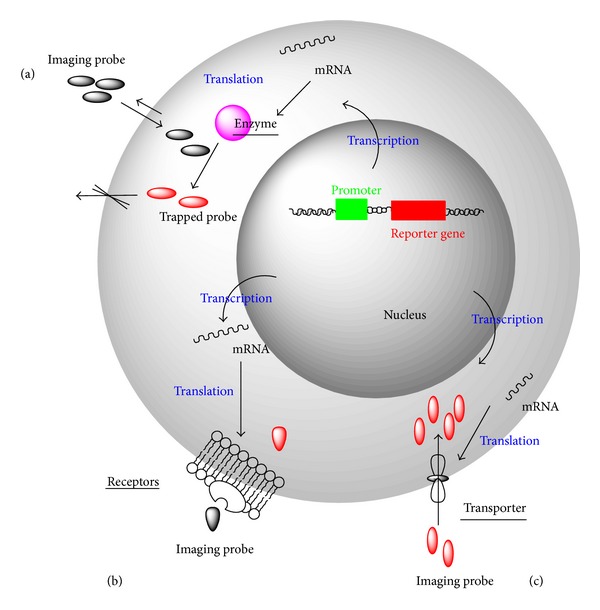
Three types of PET imaging reporter gene strategies. (a) Enzyme-based reporter gene imaging system. (b) Receptor-based reporter gene imaging system. (c) Transporter-based reporter gene imaging system.

An alternative approach is indirect imaging which can solve those problems. One example of indirect imaging that is now widely used is reporter gene imaging. Reporter gene approaches have the advantage over simple cell labeling for long-term tracking of cells because the imaging gene is passed on the cell progeny, and the imaging signal intensity is not lost through dilution by egress of the label from the cell. When a gene-marked cell dies or is phagocytosed by immune cells, the imaging signal intensity is also lost, unlike the situation with simple cell labeling in which the imaging signal intensity is not dependent on cell viability and may originate from extracellular space or from within immune scavenger cells [44]. Generally, a number of reporter genes have been developed for radionuclide imaging, including enzyme-based, receptor-based, and transporter-based (Figure 4) [45].

### 3.1. Enzyme-Based Reporter Gene Imaging System

The best known and widely used reporter gene for PET is the herpes simplex virus type 1 thymidine kinase (HSV1-tk) and its mutant form HSV1-sr39-tk. HSV1-tk expression results in the production of the enzyme HSV1-TK that phosphorylates the radiotracers such as ^124^I- or ^131^I-labeled 2′-fluoro-2′-deoxy-1-*β*-D-arabinofuranosyl-5-iodouracil (FIAU) and ^18^F-labeled 9-[4-fluoro-3-(hydroxymethyl)butyl] guanine (^18^F-FHBG), causing it to become trapped inside the cells. This modality is of particular use as the HSV thymidine kinases are not in the host tissue and, therefore, noise from nontarget tissues is minimal. In addition, because this enzyme is only expressed when the cell is living, the enzyme should mimic cell viability and disappear with cell death. 

Unfortunately, the molecular probes for the HSV1-tk enzyme (both of those based on radiolabeled uracil nucleosides and acycloguanosine derivatives) barely penetrate the intact BBB [46]. The BBB is a selective barrier formed by endothelial cells, astrocytes, basement membrane, and pericytes and neurons that are in physical proximity to the endothelium [47]. It acts as a physical barrier on account of complex tight junctions between adjacent endothelial cells, forcing most of the molecular traffic to take a transcellular route across the BBB, rather than moving paracellularly through the junctions, as in most endothelia. This effectively filters most ionized water-soluble molecules >180 Da in molecular weight [48]. Most molecular probes for HSV1-tk are based on the structure of ganciclovir, which has a molecular weight of 255 Da and only achieves a concentration in the brain of about 50% of the plasma level [49]. ^18^F-labeled acycloguanosine derivatives are heavier with extra methyl and fluoro side chains added to the ganciclovir structure.

**Figure 5:**
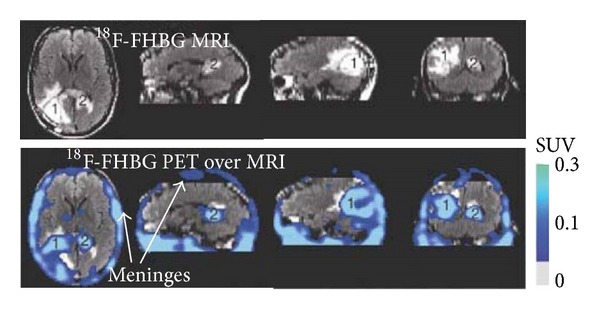
MRI and PET over MRI superimposed brain images of the patient who had been infused with autologous cytolytic T cells expressing IL-13 zetakine and HSV1-tk genes. Images were acquired approximately two hours after ^18^F-FHBG injection. The patient had a surgically resected tumor (1) in the left corner and a new nonresected tumor in the center (2), near corpus callosum of his brain. The infused cells had localized at the site of tumor 1 and also trafficked to tumor 2. ^18^F-FHBG activity is higher than the brain background at both sites. Background ^18^F-FHBG activity is low within the central nervous system due to its inability to cross the blood brain barrier. Background activity is relatively higher in all other tissues. Activity can also be observed in the meninges. The tumor 1/meninges and tumor 2/meninges ^18^F-FHBG activity ratio in this patient were 1.75 and 1.57, respectively. Whereas the average resected tumor site/meninges and intact tumor site to meninges ^18^F-FHBG activity ratio in control patients were 0.86 and 0.44, respectively.

While a number of studies have used the HSV-tk system to tag stem cells for myocardial infarction [50, 51], only several studies to date have used the HSV-tk system to image stem cells intracranially. In one particular study [52], the HSV1-tk reporter gene was used with ^18^F-FHBG probe in c17.2 murine NPCs transfected with a retrovirus and implanted intracranially in gliomaburdened rodent brains. The group found that the cells could only be visualized if they were implanted in the same hemisphere as the gliomas, as the BBB was only disrupted on that side, and this was corroborated by gadolinium-MRI. In a case report by Yaghoubi et al. [53], they detected therapeutic cytolytic T cells with ^18^F-FHBG PET in a patient with glioma. The imaging study showed above background ^18^F-FHBG signal at the site of cytolytic T cell infusions and revealed trafficking of these cells to a remote recurrent tumor in the patient's corpus callosum (Figure 5). However, it is important to note that while ^18^F-FHBG can be used to image the CNS in situations where the BBB is compromised, this substrate cannot cross an intact BBB and thus cannot detect labeled cells present in healthy brain tissue [52]. Nonetheless, this method is a promising approach for tracking stem cells in the context of brain injuries, such as stroke, given that resulting damage to the CNS typically involves the localized opening of the BBB.

Recently, Wu et al. [54] have monitored bone marrow stem cells (BMSCs) with a reporter gene system, HSV1-tk-^131^I-FIAU, in experimental MCAO rat models. The aims of their study were to investigate the feasibility of imaging HSV1-tk with ^131^I-FIAU, autoradiography, and SPECT and to investigate the optimal conditions for further imaging in vivo. Radioactivity accumulation on autoradiography and SPECT images occurred only in experimental rat models, not in controls, indicating that ^131^I-FIAU was not taken up by the normal brain and the BMSCs. Furthermore, their findings showed indirectly that experimental cerebral infarction led to the destruction of the BBB, providing the necessary condition for radionuclide imaging of the reporter gene. They concluded that the HSV1-tk-^131^I-FIAU reporter gene-probe system may be used to monitor BMSCs activity in experimental MCAO rat models. Local injection of stem cells may provide optimal means for cell transplantation and imaging with ^131^I-FIAU 24 h after injection provided peak target-to-nontarget count ratios.

The disrupted BBB has been shown in some studies to allow passage of similar probes in experimental rodent intracranial tumors, but this is not a consistent observation. Attempts to modulate the permeability of the BBB pharmacologically have been undertaken to enhance chemotherapeutic drug delivery within the brain. LeMay et al. [55] previously demonstrated that the vasodilatory bradykinin analog RMP-7 increased brain tumor permeability to ganciclovir. It remains to be investigated whether the use of osmotic disruption or RMP-7 may possibly increase the delivery of other HSV1-tk substrates across the BBB for molecular neuroimaging purposes or not. In particular, new strategies to circumvent the normal BBB or that target a blood-tumor barrier by the use of novel carrier vehicles and local vasodilation or osmotic opening all merit attention, as well as the design of newer reporter gene/probe systems tailored to molecular neuroimaging. One example of an alternative system is the xanthine phosphoribosyl transferase reporter enzyme, which has the added advantage that xanthine reporter probes can cross the BBB. The feasibility, sensitivity, and specificity of this system have already been successfully tested in an intracranial glioma modal with almost intact BBB using ^14^C-xanthine and quantitative autoradiography [56].

### 3.2. Receptor-Based Reporter Gene Imaging System

Alternative methods for imaging gene expression utilize the expression of receptor that can be targeted by a specific reporter ligand (probe). The dopamine D2 receptor (D2R) has been one of the most widely studied receptor systems with established tracers such as ^18^F-labeled fluoroethyl spiperone (FESP) [57]. Ectopic expression of the D2R as a PET reporter gene can be detected following systemic ^18^F-FESP injection. FESP binds with high affinity to the D2R, and has been shown to provide a quantitative measure of D2R expression in living animals [58]. The D2R reporter is one of the few systems which can be used for cell tracking in the brain because of the reporter probes for PET and SPECT which readily cross the intact BBB. Additionally, the D2R is encoded by endogenous genes and therefore do not elicit an immune response. However, in the brain, the presence of endogenous D2R, particularly in the striatum would provide a large background signal, and make it difficult to resolve the presence of the D2R reporter system. Another possible disadvantage is that because only one reporter probe molecule can be retained per receptor molecule, the resultant reporter probe signal may not be high enough for detection if low gene transfer occurs [59].

### 3.3. Transporter-Based Reporter Gene Imaging System

Transport proteins can be expressed in cell membranes and have high specificity for radiotracers. For example, the sodium/iodide symporter (NIS), which has been employed as a reporter gene, can be imaged with radioactive iodine, such as ^123^I for SPECT or ^124^I for PET, or other tracers, which binds to the same site [38]. However, cells that are transfected with the NIS gene do not always sufficiently retain the radioprobe due to the rapid release of radioactive iodide [60]. Additionally, it should be stressed that, like the HSV1-tk system, imaging NIS expression in the brain is not possible as the tracers do not cross the intact BBB.

 Recently, besides the above mentioned three types of reporter gene imaging systems, intracellular polymeric molecules such as melanin have also been used for reporter gene imaging. For this approach, human tyrosinase (TYR)—the key enzyme in melanin production—were introduced into cells through gene transfer; then the expressed tyrosinase catalyzes the synthesis of melanin from tyrosine precursors. Lastly, PET can be realized through introducing the melanin avid probes such as N-(2-(diethylamino) ethyl)-^18^F-5-fluoropicolinamide (^18^F-P3BZA) as a PET reporter probe. Therefore, TYR reporter gene expression can be effectively detected through melanin, combined with this reporter probe, which is also supported by results of the cell uptake experiments as well as in vivo small animal imaging studies [61].

 Reporter gene imaging can provide information on four important features: cell tracking, cell viability, cell function, and cell proliferation. However, it is still not widely used in humans because of the possible biohazard risks associated with genetic modification. PET reporter gene technology also requires stable and specific expression of a transgene and multiple exposures to ionizing radiation [62].

Using ^18^F-fluorothymidine (^18^F-FLT), a radiolabelled thymidine analog, PET has been employed to track endogenous stem cell populations. ^18^F-FLT is phosphorylated by thymidine kinase 1 during the onset of S-phase; thus, it is possible to visualize cell proliferation with PET [63]. In a healthy brain, proliferation of cells has ceased except for the SVZ and SGZ, where neural stem and progenitor cells reside. Rueger et al. [64] detected these endogenous neural stem cells in healthy rat brains. One week after stroke, ^18^F-FLT binding within the ischemic lesion, SVZ and SGZ were elevated. They demonstrated that ^18^F-FLT PET could be used not only to visualize endogenous NSCs in adult rats in vivo but also to quantify the expansion of the NSCs niche induced by pharmacological stimuli and stroke. However, ^18^F-FLT is not a neurogenesis-specific PET tracer but a proliferation marker; thus, following stroke, reactive gliosis or immune cell infiltration will be undistinguishable from neural progenitor proliferation [65].

The most commonly used radiolabeled probe to image brain activity is ^18^F-fluorodeoxyglucose (^18^F-FDG). FDG-PET has been used to detect downstream functional consequences of cell transplantation [66, 67]. After establishment of focal cerebral ischemia with the occlusion of the middle cerebral artery and cell transplantation, FDG-PET scans and neurologic function tests were performed to evaluate stem cells treatment response. Compared with the control group, higher ^18^F-FDG accumulation in the ipsilateral cerebral infarction was observed in both the induced pluripotent stem cells and the embryonic stem cells treatment groups during the 4 weeks period [66]. 

Furthermore, FDG-PET that is routinely used in the clinic for both diagnosis and posttherapy monitoring of patients already has been used to image brain metabolism after NPCs transplantation into stroke patients [68]. Following neuronal transplantation, patients were injected with 7 mCi of ^18^F-FDG, and PET imaging was performed 40 minutes later. PET images were normalized and the ^18^F-FDG uptake in the infarct and peri-infarct zones was expressed as percentage of baseline. PET scans performed 6 months posttransplantation revealed a 15% increase in ^18^F-FDG uptake at the transplant site and the ipsilateral adjacent parenchyma in 6 of 11 patients. However, it was not known if the increased FDG uptake was related to metabolic activity in the transplanted cells, host neurite outgrowth, neovascularization, improved endogenous metabolism at the transplant site, or a host inflammatory response.

## 4. Optical Imaging 

Optical imaging encompasses a variety of cell imaging modalities including fluorescence imaging and bioluminescence imaging (BLI). All of these methods involve the detection of photons emitted either by chemical oxidative processes or by external excitation of a fluorophore. In spite of their high sensitivity and superior signal-to-background ratio, all of these optical imaging modalities have poor depth penetration due to high absorption and scatter.

### 4.1. Fluorescence Imaging

In vivo fluorescence imaging utilizes organic fluorophores in the forms of fluorescent dyes such as DiD, Dil, and FDA-approved Indocyanine Green (ICG) [69]; endogenous fluorescent proteins like green fluorescent protein (GFP) and red fluorescent protein (RFP) that have been introduced to the cell by transfection or fluorochromes combined with various types of molecular probes, including targeting probes and enzyme-activatable probes [70]. Near-infrared fluorochromes that decrease absorption and enhance depth penetration are the most promising [71, 72]. Several studies have labeled stem cells with a near-infrared fluorescent dye for noninvasive optical tracking [73]. In one study, they showed that near-infrared technology was a simple and readily available technology that assured rapid semiquantitative analysis and immediate traceability of tagged hMSCs following transplantation both in vivo in anesthetized animals and ex vivo in tissue sections [71]. In another study, they demonstrated that near-infrared red (NIR) fluorescence imaging could noninvasively detect the NIR fluorescence emitted from the transplanted BMSCs engrafted in the peri-infarct neocortex through the scalp up to 8 weeks after transplantation. The results were supported by the findings on ex vivo NIR fluorescence imaging and histological analysis. Furthermore, the current results open up new opportunities for developing noninvasive NIR fluorescence imaging as a modality to track the BMSCs transplanted into the brain [74]. However, fluorescent dyes and fluorochromes have a limited half-life and are diluted out of cell division, making them not suitable for long-term cell tracking. Although genetic transduction with a fluorescent transgene can allow permanent expression of the corresponding fluorescent protein, this method runs the risk of inducing unwanted mutations in the stem cells' population during transgene process.

Currently, progress in nanotechnology has enabled us to apply biocompatible inorganic fluorescent semiconductor nanocrystals called quantum dots (QDs). QDs that consist of an inorganic core, a shell of metal, and an outer organic coating with a total diameter of 2–10 nm are becoming very important for in vivo imaging. QDs have several advantages over their organic fluorophore counterparts. For one thing, they have a broader absorption spectrum and a narrower emission spectrum, allowing multiple quantum dots with different emission energies to be imaged simultaneously with the same excitation energy [75]. Furthermore, QDs can be synthesized to the desired specifications, including size, shape, and photon emission energy. They also are more stable, have enhanced quantum yields, decreased photobleaching, and less scatter, enhancing their depth penetration [75]. Unfortunately, cell labeling with QDs would suffer from the same long-term in vivo imaging difficulties as directly labeling with regards to dilution during cell proliferation. Nevertheless, quantum dots have been successfully used for stem cells tracking. One study has showed the labeling of neural stem and progenitor cells with quantum dots by electroporation and ultrasound-guided biomicroscopy [76]. Especially QDs with an emission spectra in the far red or near infrared have much longer wavelengths that allow easy penetration of tissue, including bone and skin [74]. Near-infrared emitting QDs have a potential for in vivo tracking of cells within the brain, as demonstrated by Kawabori et al. [77]. 

### 4.2. Bioluminescence Imaging

BLI is the most well studied of the various optical imaging modalities with regards to stem cells imaging in the brain. Bioluminescence involves transducing a reporter gene, which codes for firefly luciferase or renilla luciferase, into the stem cells. When the luciferase enzyme reacts with its substrate d-luciferin or coalenterazine, it emits photons which can then be detected and quantified by a charge-coupled device camera system [78]. For some luciferases, like the firefly luciferase, the light emitting reaction is dependent on ATP, thus allowing its detection as a viability marker of the luciferase expressing cells. In addition, the light emission is directly proportional to the number of cells, which further permits cell quantification [52, 79, 80]. Using BLI, cell tracking can be performed over long periods of time since the luciferase gene is stably integrated into the genome, and luciferase expression is preserved during proliferation or differentiation so that all cell progeny express luciferase without dilution [81]. Therefore, BLI has already been used to study in vivo stem cells migration, viability and immunogenicity, and tumorigenicity in small animal studies [82–85]. However, all luciferase emissions are in the visible spectrum, which is prone to scattering and absorption in tissue [43]. Even firefly luciferase, with a comparatively long peak wave-length of 562 nm, is limited to the use in small animals due to a maximum penetration of 3 cm in tissue [80]. Moreover, for BLI, requirement of engineering cells runs the risk of introducing unwanted mutations. Therefore, BLI is not feasible for clinical translation.

Nonetheless, BLI has been shown to be an effective method to study in vivo stem cells migration and viability in several studies. Kim et al. [81] used BLI to track murine C17.2 NPCs after transplantation in a murine model of stroke. C17.2 NPCs, stably transfected with firefly luciferase, were serially imaged through intact skull and skin by bioluminescence imaging over 2 to 3 weeks in nu/nu mice with closed-vessel middle cerebral artery infarcts, followed by contralateral intraparenchymal or intraventricular injections of NPCs. NPCs migrated to the site of infarct from the contralteral parenchyma, crossing the midline at 7 days. In control animals without infarcts, NPCs remained at the site of administration. Intraventricular cell administration resulted in a wide distribution of cells, including the site of infarct. The findings were confirmed with histology.

**Figure 6:**
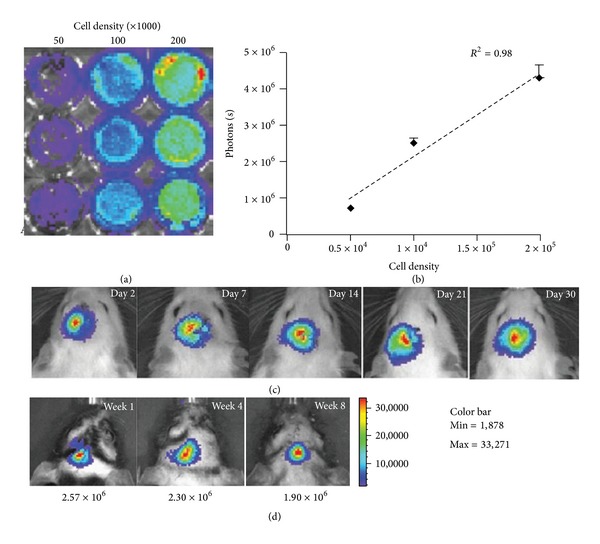
In vitro and in vivo bioluminescence imaging (BLI) of the hNSCs. (a) In vitro  imaging analysis of genetically engineered hNSCs shows increasing fluc activity with cell density and a linear correlation (*R* ^2^ = 0.98). (b) Data are representative of three independent experiments performed in triplicate. Representative BLI of stroke-lesioned rats transplanted with the hNSCs and monitored for (c) 4 weeks and (d) 8 weeks posttransplantation survival times. Quantitative analysis of the fluc activity in these animals shows a stable BLI signal, which suggests the survival of the grafts and the nonproliferative property of the hNSCs. Color scale bar is in photon/s/cm^2^/sr.

In another study, BLI was used to track the fate of grafted hNSCs in the stroke-damaged rat brain [9]. Daadi et al. labeled hNSCs with SPIO, as well as firefly luciferase and enhanced GFP coded by the so-called double fluorescence transgene. The cells were then introduced into a stroked mouse model and were followed for 2 months in vivo  using BLI and MRI. Longitudinal quantitative analysis shows a stable BLI signal, which suggested the survival and nontumorigenic property of the hNSCs. In addition, they demonstrated that the gene transduction process did not alter the physiology of the cells and allowed for normal hNSCs differentiation into neurons, astrocytes, and oligodendrocytes. When the cells were introduced in vivo, they migrated as expected towards the lesion. These were confirmed by immunohistology and electrophysiology in vitro and in vivo. In addition, dose-BLI signal relationships in vitro were maintained in vivo and were corroborated by similar cell numbers as extrapolated from the dose-magnetic resonance signal relationship (Figure 6) [9].

Several groups have used BLI to follow also endogenous neurogenesis in health [86, 87] and disease. Reumers et al. [86] labeled cells in the SVZ by injecting lentiviruses which stably expressed firefly luciferase in wild type mice. Bioluminescence was recorded with high sensitivity to follow the migration of infected neuroblasts from the SVZ to the olfactory bulb. Another method for bioluminescence of migration of endogenous neural stem cells was adopted by Couillard-Despres et al. [87], who created a transgenic mouse, which expressed luciferase under the control of the doublecortin promoter, a protein expressed by migratory neuroblasts of the SVZ. Moreover, the use of a neuronal precursor-specific promoter to drive luciferase expression guaranteed that the bioluminescence was neurogenesis-specific. Using these transgenic mice, an increase of SVZ cell proliferation after cerebral ischemia could be followed with OI. These observations were in line with earlier invasive studies where stroke resulted in increased proliferation of the ipsilateral SVZ and more lateral migration of these early neurons towards the lesion [88, 89].

## 5. Multimodality Molecular Imaging

Because each of the aforementioned imaging technologies has unique advantages and disadvantages, researchers have developed genes, probes, contrast agents, and detectors that are compatible with different imaging modalities. Multimodality molecular imaging combined of two or more modalities offers the possibility to minimize the potential drawbacks of using each imaging modality alone, to integrate modality-specific strengths, and to gain a more complete picture of graft behavior. For example, a complementary use of optical imaging or PET for high sensitivity and specificity together with MRI for high spatial and temporal resolution has found applications of cell tracking following ischemia stroke.

The development of multimodality noninvasive imaging reporter genes will allow us to choose appropriate imaging technologies to meet the demands of specific molecular biologic problems. Several strategies based on the combined expression of multiple genes have been reported. The bicistronic approach for linking two genes involves the incorporation of an internal ribosomal entry site sequence between the two genes. Both genes are then transcribed into a single mRNA and translated into two different proteins. The second strategy uses fusion gene vectors, whereby the two genes are connected in such a way that their coding sequences are in the same reading frame. The third mean uses bidirectional transcription, as was used to coexpress the HSV1-sr39-tk and D2R genes in a bidirectional manner with centrally located tetracycline-responsive promoter elements [90].

Recently, the triple-fusion reporter gene expresses a triple-fusion protein, which contains coding regions for red or green fluorescent protein, luciferase, and HSV1-TK enzyme. This fusion reporter offers the possibility of using the particular imaging technique that best suits the application; fluorescence for studying individual cells or for cell sorting, bioluminescence for high sensitivity in vivo imaging in small animals, and PET or SPECT when quantitative accuracy is important, or for the translation to humans. In one study of Cao et al. [50], they used BLI and ^18^F-FHBG PET to moniter Murine ESCs stably transduced with a lentiviral vector carrying a novel triple-fusion reporter gene that consisted of firefly luciferase, monomeric red fluorescence protein, and truncated thymidine kinase (fluc-mrfp-ttk). They demonstrated how that a novel molecular imaging platform could be used to monitor the kinetics of stem cells survival, proliferation, migration, and ablation of teratoma sites. Furthermore, they showed that expression of reporter genes and their interaction with reporter probes did not adversely affect ESCs viability, proliferation, and differentiation.

**Figure 7:**
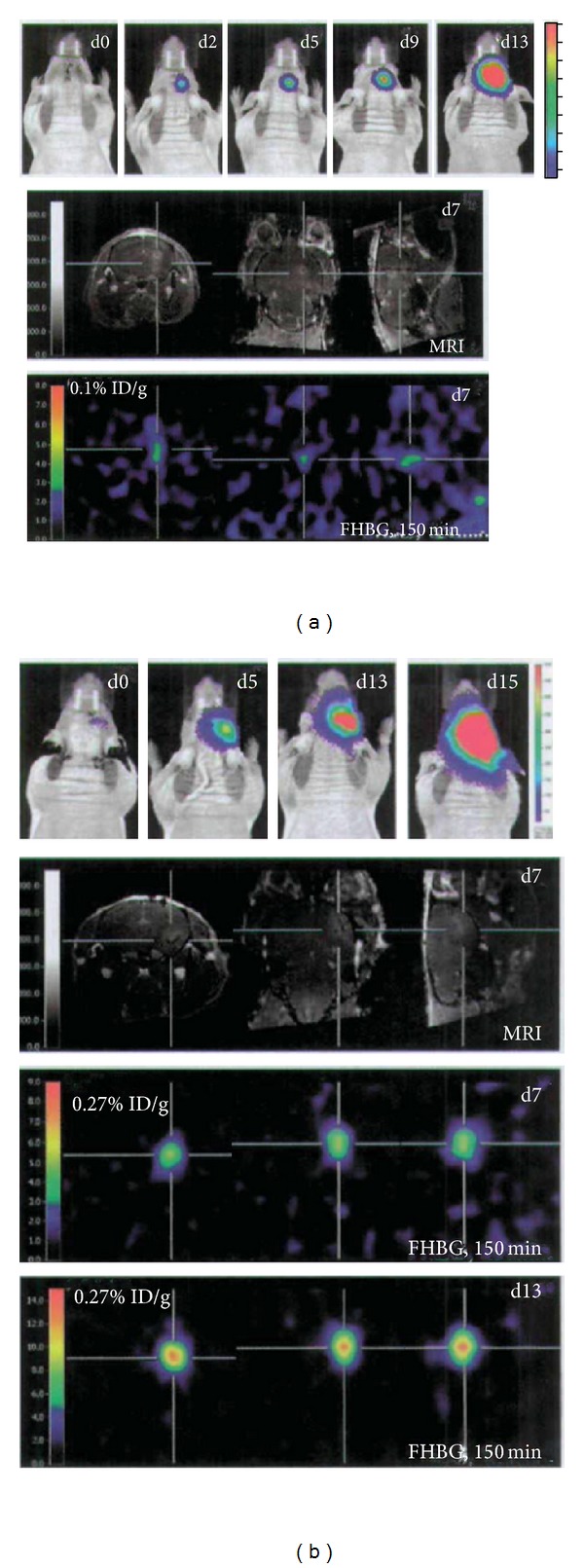
Multimodal imaging of neural progenitor cells (NPCs). Displayed are data from one representative animal with injection of NPCs within a preestablished glioma (a) and from one representative animal with injection of NPCs mixed with glioma cells (b). Upper row, BLI signal over the brain at 0, 2, 5, 9, and 13 days, respectively 0, 5, 13, and 15 days postimplantation. Second row, Gadolinium-enhanced T1 MRI at 7 days posttransplantation. The cross focuses on the growing glioma. Lower rows, ^18^F-FHBG PET image at 7 and 13 days posttransplantation, matched to the MRI displayed in the second row. After 150 minutes of tracer incubation, specific ^18^F-FHBG accumulation is visible at the site of glioma proliferation in both injection paradigms ((a) and (b)). The BLI signal intensity increases between days 5 and 15 in the mixed injection paradigm which is also reflected in an increased ^18^F-FHBG uptake between days 7 and 13 (b).

In another study, Waerzeggers et al. [52] demonstrated that multimodal imaging of the fate of NPCs inoculated into the rodent brain was feasible using reporter gene technology (Figure 7). BLI served the higher sensitivity for detecting luc-expressing cells, especially in conditions in which the BBB was intact. In conditions in which the BBB was disrupted, ^18^F-FHBG-PET also served for localization of tk-expressing cells. MRI experiments after gadolinium diethylenetriamine pentaacetic acid (Gd-DTPA) administration were performed for evaluating the integrity of the BBB. They demonstrated that sequential, noninvasive multimodality imaging may serve as a safety switch for early detection of aberrant cell migration and excessive cell proliferation.

Multimodality molecular imaging enables real time longitudinal monitoring of infarct location, size, and transplant survival. In one study, Daadi et al. [13] used MRI and PET to track the infarct evolution, tissue repair, and the fate of grafted cells. They genetically engineered embryonic stem cell-derived NSCs with a triple fusion reporter gene consisting of HSV-tk, luciferase, and GFP for multimodality molecular imaging and SPIO labeled for MRI. The infarct size, fate, and function of grafted cells were tracked in real time for 3 months using MRI and PET. They reported that grafted NSCs reduced the infarct size in animals with less than 0.1 cm³ initial infarct in a dose-dependent manner, while a larger stroke was not amenable to such beneficial effects. PET imaging revealed increased metabolic activity in grafted animals and visualized functioning grafted cells in vivo. Immunohistopathological analysis demonstrated that, after 3-month survival period, grafted NSCs dispersed in the stroke-lesioned parenchyma and differentiated into neurons, astrocytes, and oligodendrocytes. Longitudinal multimodal imaging provides insights into time course dose-dependent interactions between NSCs grafts and structural changes in infracted tissue [13]. 

Instead of using multiple genes for multimodality imaging, human TYR was evaluated as a stand-alone reporter gene for in vitro and in vivo photoacoustic imaging (PAI), MRI, and PET [61]. TYR is introduced into cells through gene transfer; then the expressed tyrosinase catalyzes the synthesis of melanin from tyrosine precursors. The resulting gene product, melanin, can then be imaged by three modalities. Firstly, PAI can be achieved because melanin has a broad optical absorption spectrum and is; therefore, an excellent agent for photoacoustic effect which in turn can be utilized to perform PAI. Secondly, melanin has the ability to chelate metal ions (e.g., Fe^3+^) which provides contrast for MRI. Lastly, PET can be realized through introducing the melanin avid probes benzamide analogs such as ^18^F-P3BZA as a PET reporter probe. In their study, they demonstrated that TYR is a highly promising reporter gene system for multimodality molecular imaging in biomedical research.

Another type of multimodality imaging is multimodal contrast agents, which integrate multiple properties within one nanoparticle to allow its detection with several imaging techniques [91]. Magnetic quantum dots based on highly fluorescent QDs combined with magnetic nanoparticles or ions form an exciting class of new materials for bioimaging [92]. With two functionalities integrated in a single nanoparticle, a sensitive contrast agent for two very powerful and highly complementary imaging techniques including fluorescence imaging and magnetic resonance imaging is obtained. There are four different approaches to integrate the fluorescence and magnetic properties in a single nanoparticle. The first type of particles is created by the growth of heterostructures in which a QD is either overgrown with a layer of a magnetic material or linked to magnetic nanoparticles. The second approach involves doping of paramagnetic ions into QDs. A third option is to use silica or polymer nanoparticles as a matrix for the incorporation of both QDs and magnetic nanoparticles. Finally, it is possible to introduce chelating molecules with paramagnetic ions (e.g., Gd-DTPA) into the coordination shell of the QDs. Although this type or design of particle is the most advantageous or promising for bioapplications, issues remain that must be resolved; for example, the toxicity and the circulation half-time of the particles and the mechanisms by which they are cleared (kidney, liver, or elsewhere) [92]. 

## 6. Conclusion

Stem cells therapy for the stroke can only be taken to the clinic, if safety and efficacy of the transplanted cells can be guaranteed. Therefore, it is important to refine and optimize tracking techniques, so that they can be used in a clinical setting. Currently, every imaging technique used for cell tracking has merits and defects. Finally, future efforts should focus on multimodality imaging approaches, which may minimize the potential drawbacks of using each imaging modality alone, as it is possible that a tailored combination of two or more techniques may provide the most ideal information profile for clinical applications.

## Abbreviations

BBB: Blood-brain barrier
MRI: Magnetic resonance imaging
OI: Optical imaging
GRID: Gadolinium rhodamine dextran
hNSCs: Human neural stem cells
SPIO: Superparamagnetic iron oxide particles
MPIO: Micron-sized paramagnetic particles
USPIO: Ultrasmall superparamagnetic iron oxide particles
MCAO: Middle cerebral artery occlusion
ESCs: Embryonic stem cells
GFP: Green fluorescent protein
SVZ: Subventricular zone
SGZ: Subgranular zone
NPCs: Neural progenitor cells
SPECT: Single photon-emission computed tomography
PET: Positron emission tomography
HSV1-tk: Herpes simplex virus type 1 thymidine kinase
FIAU: 2′-fluoro-2′-deoxy-1-*β*-D-arabinofuranosyl-5-iodouracil
^18^F-FHBG: ^18^F-labeled 9-[4-fluoro-3-(hydroxymethyl) butyl] guanine
D2R: Dopamine D2 receptor
FESP: Fluoroethylspiperone
NIS: Sodium/iodide symporter
TYR: Tyrosinase
^18^F-P3BZA: N-(2-(diethylamino) ethyl)-^18^F-5-fluoropicolinamide
^18^F-FLT: ^18^F-fluorothymidine
^18^F-FDG: ^18^F-fluorodeoxiglucose
BLI: Bioluminescence imaging
QDs: Quantum dots
ICG: Indocyanine green
RFP: Red fluorescent protein
NIR: Near-infrared red
Gd-DTPA: Gadolinium diethylenetriamine pentaacetic acid.
